# A Redox-Controllable Molecular Switch Based on Weak Recognition of BPX26C6 at a Diphenylurea Station

**DOI:** 10.3390/molecules20021775

**Published:** 2015-01-22

**Authors:** Jia-Cheng Chang, Chien-Chen Lai, Sheng-Hsien Chiu

**Affiliations:** 1 Department of Chemistry, National Taiwan University, No. 1, Sec. 4, Roosevelt Road, Taipei 10617, Taiwan; E-Mail: f99223182@ntu.edu.tw; 2 Institute of Molecular Biology, National Chung Hsing University, Taichung 402, Taiwan; E-Mail: lailai@dragon.nchu.edu.tw

**Keywords:** BPX26C6, molecular switch, redox, rotaxane, urea

## Abstract

The Na^+^ ion–assisted recognition of urea derivatives by BPX26C6 has allowed the construction of a redox-controllable [2]rotaxane-type molecular switch based on two originally very weakly interacting host/guest systems. Using NOBF_4_ to oxidize the triarylamine terminus into a corresponding radical cation attracted the macrocyclic component toward its adjacent carbamate station; subsequent addition of Zn powder moved the macrocyclic component back to its urea station.

## 1. Introduction

**Figure 1:**
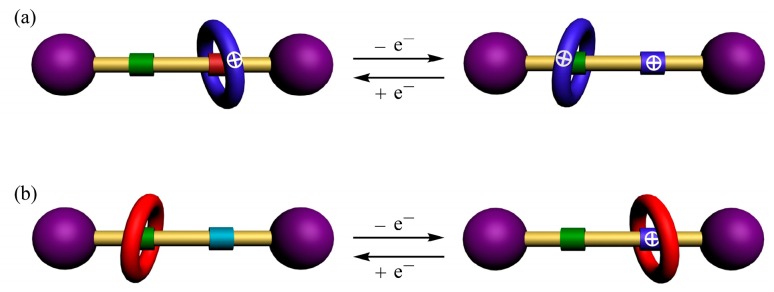
Cartoon representations of (**a**) repulsion- and (**b**) attraction-type redox-controllable [2]rotaxane-based molecular switches.

Because of the many potential applications of rotaxane-based molecular switches (e.g., in material transportation [1,2], molecular memory [3], sensing [4,5], and gelation [6,7]), there is keen interest in finding new recognition units for the assembly of these functional interlocked molecules and in developing new methods for their reversible operation [8,9,10,11,12,13,14,15]. Although the basic switching cycle in a simple bistable [2]rotaxane-type molecular switch requires only an appropriately large and invertible difference in stabilization energy for the complexation of the macrocyclic component at the two stations in the dumbbell-shaped component, in practice one of these stations must have sufficiently strong binding affinity to the macrocycle to ensure efficient synthesis of the [2]rotaxane in the first place. Thus, guest species that have very weak binding affinity to macrocycles are frequently ignored as components in such molecular switches: they cannot serve as primary recognition units to facilitate the synthesis of the rotaxanes and they cannot be used as secondary recognition units in the presence of a much more tightly binding guest to allow successful switching. Previously, we demonstrated that Na^+^ ions can template the threading of a urea or amide unit through the cavity of BPX26C6; we then applied this recognition system to construct a pH- and Na^+^-controllable molecular switch based on this macrocycle and two of its very weakly associating guests: a tertiary ammonium ion and a diphenylurea [16,17,18]. We suspected that such a concept might also be used to prepare a redox-controllable molecular switch. Although applying a redox process to induce charge repulsion between a cationic interlocked macrocyclic component and a primary recognition unit is an efficient and frequently used strategy for operation of redox-controllable molecular switches [19], a neutral rotaxane that features weak interactions between its macrocyclic component and its primary recognition unit might allow for a different operation strategy—one in which the oxidation process increases the force of attraction between the macrocyclic component and its secondary station, thereby, facilitating switching (Figure 1) [20,21,22,23,24]. Herein, we report a redox-controllable rotaxane-based molecular switch having such a design; its interlocked BPX26C6 component can be moved away from (or onto) the diphenylurea station when using NOBF_4_ (or Zn powder) to oxidize (or reduce) the triarylamine unit.

## 2. Results and Discussion

**Scheme 1:**
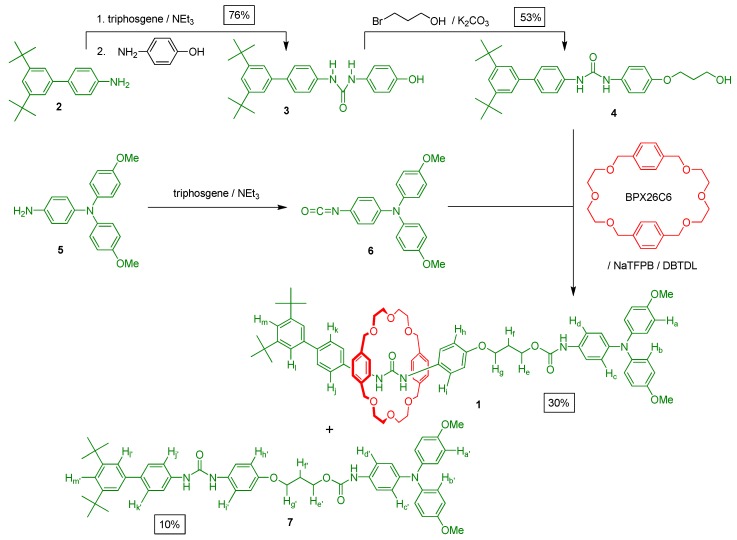
Synthesis of the [2]rotaxane **1** and the dumbbell-shaped molecule **7**.

We designed the [2]rotaxane **1** with the expectation that, in its neutral state, the interlocked BPX26C6 component would encircle the diphenylurea station exclusively, due to the affinity of this pair being higher than that of a BPX26C6/carbamate system [18]. Oxidation of the triarylamine unit into its corresponding radical cation would then drive the migration of the macrocyclic component from the diphenylurea station to the carbamate unit, due to the positive charge generated on the nitrogen atom of the triarylamine motif enhancing the strength of any possible [N–H∙∙∙O] and [C–H∙∙∙O] hydrogen bonds and π-stacking interactions between the BPX26C6 unit and the carbamate station and its adjacent aromatic ring. We synthesized the [2]rotaxane **1** in four steps from the aniline derivative **2** (Scheme 1). First, we converted **2** into the corresponding isocyanate by reacting it with triphosgene; treatment of this isocyanate with 4-aminophenol afforded the urea derivative **3**. Next, reaction of the urea **3** with 3-bromo-1-propanol under basic conditions provided the alcohol **4**. Finally, we mixed the alcohol **4** with BPX26C6 and NaTFPB in CH_2_Cl_2_ to generate the corresponding [2]pseudorotaxane, which we reacted with the isocyanate **6** (itself derived from the triarylamine **5**) in the presence of dibutyltin dilaurate (DBTDL); after column chromatography, we isolated the [2]rotaxane **1** and the dumbbell-shaped molecule **7** in yields of 30% and 10%, respectively.

**Scheme 2:**
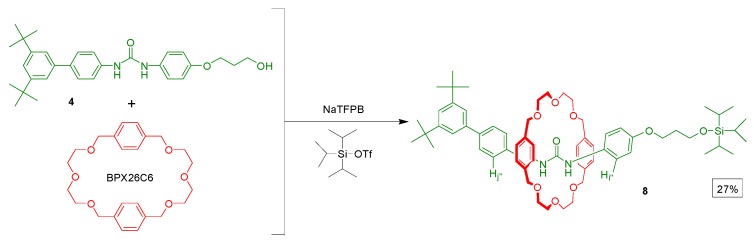
Synthesis of the [2]rotaxane **8**.

**Figure 2:**
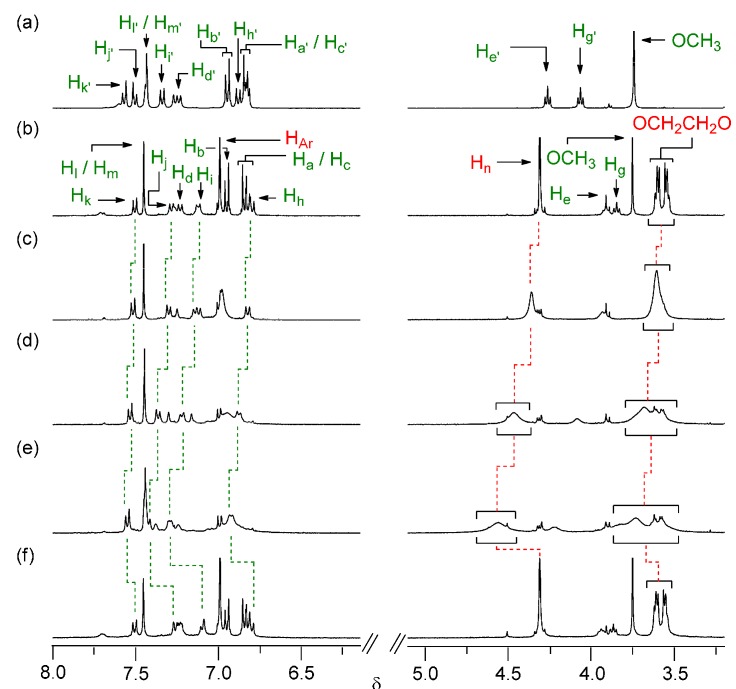
^1^H-NMR spectra (400 MHz, CD_3_CN, 298 K) of (**a**) the dumbbell-shaped molecule **7**, (**b**) the [2]rotaxane **1**, and (**c**–**f**) the solutions obtained after additions of (c) 0.5, (d) 1.0, and (e) 1.5 equiv of NOBF_4_ to the solution in (b) and (f) excess Zn powder to the solution in (e).

With the [2]rotaxane **1** in hand, we wished to confirm its preference for the interlocked BPX26C6 macrocyclic component to encircle the diphenylurea station, rather than the carbamate one. As a reference compound, we synthesized the [2]rotaxane **8** by adding triisopropylsilyl trifluoromethane-sulfonate to a mixture of the alcohol **4**, NaTFPB, and BPX26C6 (Scheme 2). 2D COSY and NOESY experiments allowed us to identify most of the signals in the ^1^H-NMR spectrum of the [2]rotaxanes **1** (Figure 2) and **8** in CD_3_CN at 298 K. Because the diphenylurea station of the dumbbell-shaped molecule **7** is not encircled by a BPX26C6 unit, and if we assume that the interlocked BPX26C6 component always resides at the diphenylurea station in the [2]rotaxane **8**, then we could estimate the preference for the macrocyclic unit residing about the diphenylurea station (over the carbamate unit) in the [2]rotaxane **1** simply by considering the chemical shifts of its urea-adjacent aromatic protons in terms of molar fraction averages of the corresponding signals of the dumbbell-shaped molecule **7** and the [2]rotaxane **8**. Using the aromatic protons in dumbbell **7** (H_i'_ and H_j'_) and rotaxane **8** (H_i''_ and H_j''_) as references, we calculated that, in CD_3_CN at 298 K, the interlocked BPX26C6 unit in the [2]rotaxane **1** prefers to reside at the diphenylurea center, rather than the carbamate station, in a 61:39 distribution ratio (∆*G*° = 0.26 kcal/mol).

A small switching ratio is all that is required when developing switches that can be used in such applications as sensors, colorimetric indicators, and logic gates [25]. Therefore, although the [2]rotaxane **1** does not behave as an “all-or-nothing” molecular switch [26], it might still have the ability to perform unique functions if it were to display suitable redox-based switching behavior.

To avoid possible disturbance from metal ion chelation during the translocation of the BPX26C6 component of the [2]rotaxane **1**, we chose NOBF_4_ as the oxidant to initiate the switching process [27,28]. The addition of NOBF_4_ to a CD_3_CN solution of the [2]rotaxane **1** resulted in significant downfield shifts of the signals of the protons of the aromatic ring adjacent to the urea station, returning them to positions similar to those observed in the ^1^H-NMR spectrum of the free dumbbell-shaped molecule **7** (Figure 2), suggesting that the BPX26C6 unit no longer encircled the urea station. The significant broadening of the signals for the xylyl and ethylene glycol units of BPX26C6 after oxidation, presumably due to the paramagnetism of the radical cation, suggested migration of the macrocyclic component to a position encircling the carbamate station. We observed negligible shifts of the signals in the ^1^H-NMR spectra after the addition of more than 1.5 equiv of NOBF_4_, implying that the triarylamine unit was almost completely oxidized under these conditions.

**Figure 3:**
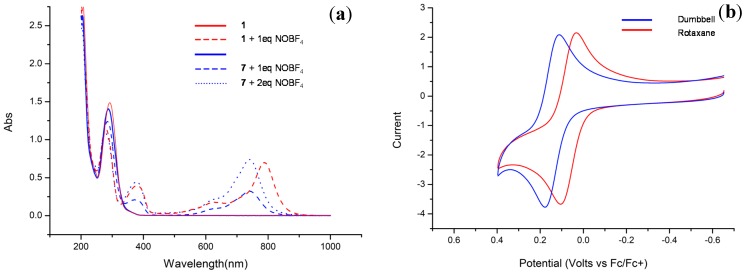
(**a**) Electronic absorption spectra of the [2]rotaxane **1** and the dumbbell-shaped molecule **7** in CH_3_CN (0.3 mM) in the presence of increasing amounts of the oxidizing agent NOBF_4_; (**b**) Cyclic voltammograms of the [2]rotaxane **1** (0.5 mM) and the dumbbell-shaped molecule **7** (0.5 mM) in CH_3_CN; supporting electrolyte: NBu_4_PF_6_ (0.1 M); scan rate: 100 mV·s^−1^.

The UV spectrum of the dumbbell-shaped molecule **7** featured an absorption band at 742 nm after oxidation mediated by 1 equiv of NOBF_4_; the corresponding signal in the spectrum of the [2]rotaxane **1** was more intense and red-shifted to 789 nm after oxidation to the corresponding radical cation by the same amount of NOBF_4_ (Figure 3a). Addition of a second equiv of NOBF_4_ to the CH_3_CN solution of the threadlike molecule **7** increased the intensity of its absorption at 742 nm to a level similar to that of the signal at 789 nm in the spectrum of the equimolar mixture of the [2]rotaxane **1** and NOBF_4_. We suspect that the differences between these long-wavelength UV absorptions for the triarylamine radical cations of the dumbbell-shaped molecule **7** and the [2]rotaxane **1** were due mostly to the presence of the BPX26C6 component in the latter; that is, the macrocyclic component stabilized the radical cation by encircling its adjacent aromatic and carbamate units. As expected, we observed one-electron oxidation processes during cyclic voltammetry (CV) of both the [2]rotaxane **1** and the dumbbell-shaped molecule **7** at potentials of less than +0.4 V. The oxidation peak potential in the cyclic voltammogram of the [2]rotaxane **1** in CH_3_CN (0.5 mM) appeared, however, at 105 mV* versus* Fc/Fc^+^—approximately 73 mV lower than that of the dumbbell-shaped molecule **7** under similar conditions (Figure 3b). This result suggests that oxidation of the triarylamine motif in the [2]rotaxane **1** is easier than that in the dumbbell-shaped molecule **7**—again presumably as a result of the encircling BPX26C6 unit stabilizing the radical cationic center (and its adjacent carbamate station), consistent with our observation in the ^1^H-NMR spectra of migration of the BPX26C6 component from the urea station toward the radical cationic motif. The higher oxidation potential of the dumbbell-shaped molecule **7** relative to that of the [2]rotaxane **1** in CD_3_CN might also explain the weaker UV absorption at 742 nm of the equimolar (0.3 mM) mixture of the dumbbell-shaped molecule **7** and the oxidant NOBF_4_, relative to the signal at 789 nm in the spectrum of the [2]rotaxane **1** and NOBF_4_ at the same concentration.

**Figure 4:**
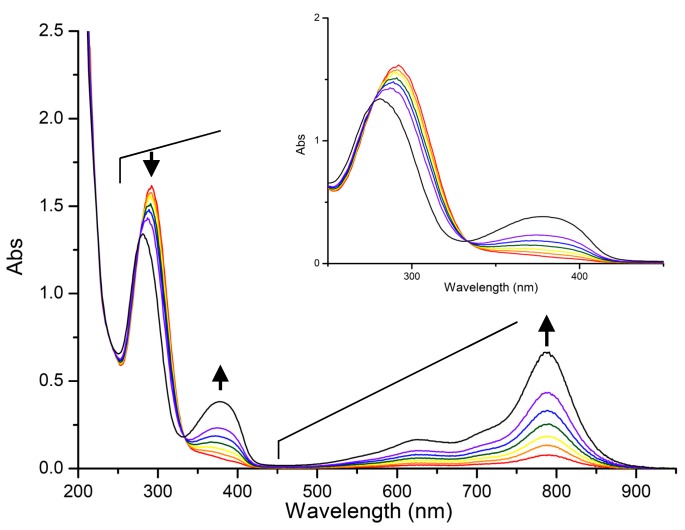
*In situ* electronic absorption spectra (0.1 M NBu_4_PF_6_ in CH_3_CN; room temperature) of the [2]rotaxane **1** (0.3 mM) at an oxidation potential of 700 mV (*vs.* Ag/AgCl) recorded over 2, 4, 6, 10, 20, 40, and 120 s, respectively

**Scheme 3:**
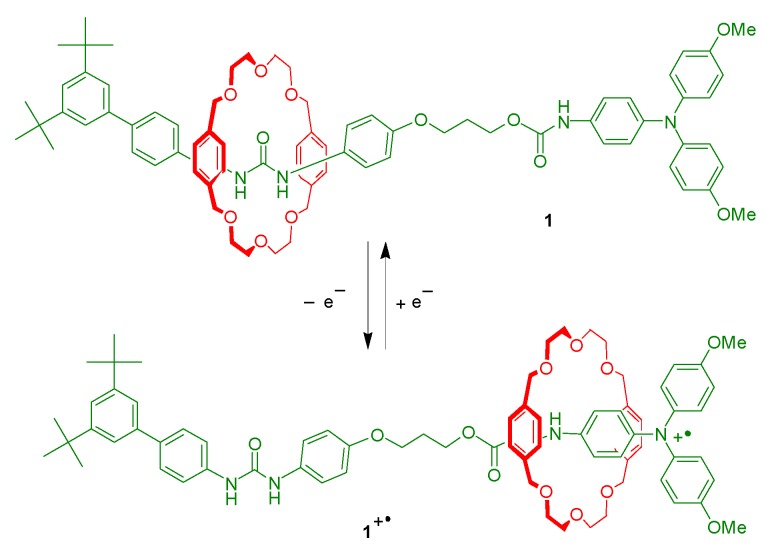
Redox-based switching of the [2]rotaxane **1**.

**Figure 5:**
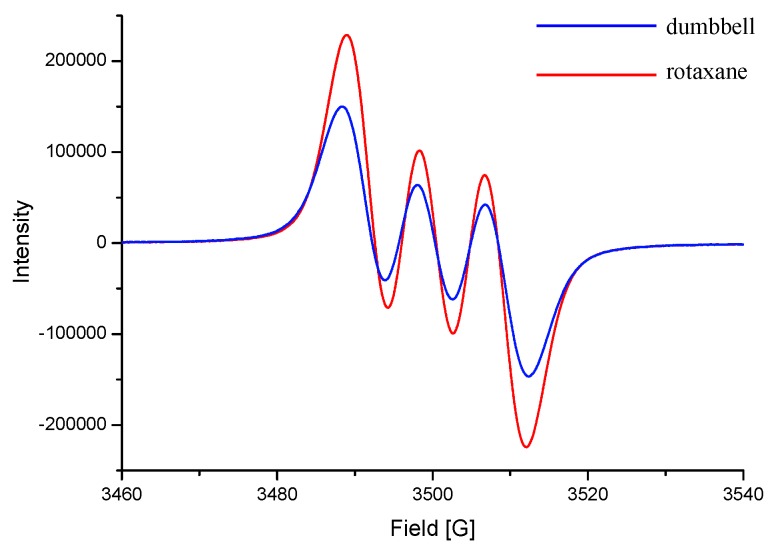
EPR spectra (CH_3_CN, 298 K) of equimolar (3 mM) mixtures of NOBF_4_ and the [2]rotaxane **1** (red line) and the dumbbell-shaped molecule **7** (blue line). Dumbbell rotaxane.

*In situ* absorption analysis of the [2]rotaxane **1** at a potential at 700 mV revealed the growth, over a period of time, of a new absorption band at 789 nm (Figure 4). The isosbestic points at 277 and 333 nm in the absorption spectra reveal that the oxidation process involved the conversion of only two species, consistent with our proposed switching mechanism (Scheme 3). The signal in the EPR spectrum of an equimolar (3 mM) solution of the [2]rotaxane **1** and NOBF_4_ was slightly upfield-shifted and more intense than that of the dumbbell-shaped molecule **7** in the presence of the same oxidant at the same concentration (Figure 5). These unresolved three-line spectra are similar to those reported previously for triarylamine analogues, suggesting that *N*-centered charge localization occurred in our system [29,30]. The shift of the EPR signal in the spectrum of the [2]rotaxane **1** presumably resulted, in part, from the interaction between the BPX26C6 unit and the radical cationic motif; we suspect, however, that because this peak migration was only slight, the relatively sterically inaccessible N-centered radical did not interact significantly with the interlocked BPX26C6 moiety. Thus, we hypothesize that the carbamate unit and its adjacent aromatic ring comprised the site at which the BPX26C6 component resided in the oxidized [2]rotaxane **1^+•^**. The weaker EPR absorption for the equimolar solution of the dumbbell-shaped molecule **7** and NOBF_4_, relative to that for the [2]rotaxane **1**/NOBF_4_ mixture, implies a lower concentration of triarylamine radical cations generated in the former case, consistent with our observation of a relatively weaker UV absorption intensity at 742 nm, due to the oxidation potential of the triarylamine motif in the dumbbell-shaped molecule **7** being higher than that of **1**. 

**Figure 6:**
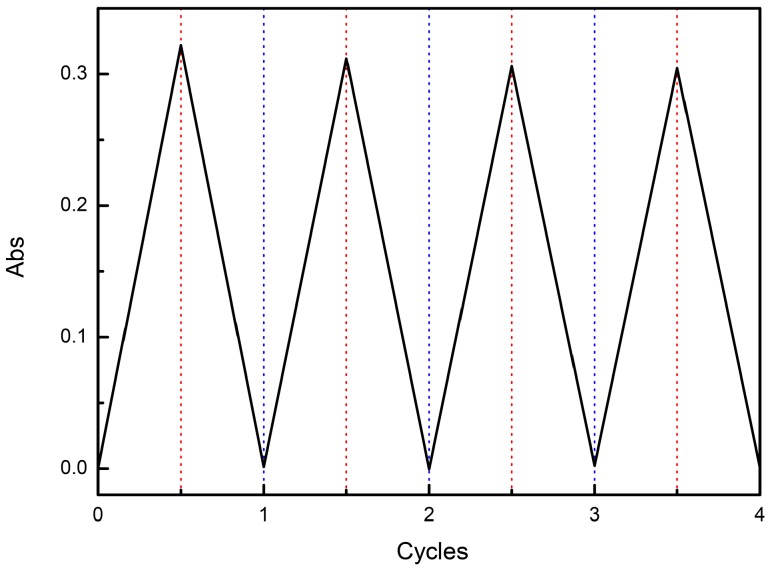
Redox switching of the [2]rotaxane **1** in CH_3_CN (0.3 mM), monitored at 829 nm using UV spectroscopy. Each switching cycle involved sequential addition of NOBF_4_ (70 µg) and Zn powder (20 mg), followed by filtration.

Having proven that oxidation of the triarylamine motif in the [2]rotaxane **1** can be achieved both chemically and electrochemically, with resulting translocation of the interlocked BPX26C6 component from the urea station to the carbamate one, we investigated the reverse process of reducing the radical cation back to its neutral state. After adding an excess of Zn powder to a solution of the [2]rotaxane **1** and NOBF_4_ (1:1.5), Figure 2f reveals a ^1^H-NMR spectrum that was similar to that of the [2]rotaxane **1** prior to adding any additives, suggesting that the majority of interlocked BPX26C6 components had returned to the diphenylurea stations after the triarylamine radical cations had been reduced to their neutral state by the Zn powder. Thus, the [2]rotaxane **1** behaves as a redox-controllable molecular switch, in which the interlocked BPX26C6 component can, after the addition of NOBF_4_, depart the diphenylurea station to encircle the carbamate station adjacent to the newly formed radical cation, and then, upon the addition of excess Zn powder, return to the urea station. We demonstrated the reproducibility and stability of this redox switch through four complete cycles of the sequential addition of NOBF_4_ and Zn powder to a CH_3_CN solution of the [2]rotaxane **1**, monitored at 829 nm using UV spectroscopy (Figure 6).

## 3. Experimental Section

### 3.1. General Information

All glassware, syringes, needles and stirrer bars were oven-dried prior to use. Unless otherwise indicated, all reagents were obtained from commercial sources. Anhydrous MeCN and CH_2_Cl_2_ were obtained by distillation from CaH_2_ under N_2_. Reactions were conducted under N_2_ atmospheres. Thin-layer chromatography (TLC) was performed on Merck 0.25 mm silica gel (Merck Art. 5715, Merck, Darmstadt, Germany) and column chromatography was undertaken over Kieselgel 60 (Merck, 70–230 mesh). Melting points are determined without correction by Fargo MP-2D melting point apparatus (Fargo, Taipei, Taiwan).

*Typical Procedure for Transforming the Amine* **2*** into the Corresponding Isocyanate.* A THF solution (25 mL) of triphosgene (263 mg, 0.886 mmol) was added to a solution of **2** (500 mg, 1.78 mmol) and Et_3_N (1.02 mL, 7.30 mmol) in THF (25 mL) at 0 °C and then the mixture was stirred at room temperature for 3 h. The solids were filtered off and the filtrate concentrated under reduced pressure to afford a yellow liquid (492 mg, 90%). The crude isocyanate was used directly without further purification.

*Urea Derivative* **3***.* A solution of the isocyanate [transformed from 500 mg (1.78 mmol) of **2**] in THF (25 mL) was added to a solution of 4-aminophenol (175 mg, 1.60 mmol) in THF (25 mL) and then the mixture was stirred at room temperature for 16 h. The solvent was evaporated under reduced pressure and the residue purified chromatographically (SiO_2_; EtOAc/hexanes, 2:8); washing of the chromatographed material with CH_2_Cl_2_ (30 mL) afforded a white solid (506 mg, 76%). M.p. 197–199 °C; ^1^H-NMR (400 MHz, CD_3_COCD_3_): δ = 1.38 (s, 18H), 6.78 (d, *J* = 8.8 Hz, 2H), 7.35 (d, *J* = 8.8 Hz, 2H), 7.43–7.50 (m, 3H), 7.56 (d, *J* = 8.8 Hz, 2H), 7.62 (d, *J* = 8.8 Hz, 2H), 7.85 (s, 1H), 8.04 (s, 1H), 8.09 (s, 1H); ^13^C-NMR (100 MHz, CD_3_COCD_3_): δ = 31.9, 35.5, 116.2, 119.9, 121.6, 121.8, 122.3, 128.2, 132.4, 136.8, 140.2, 141.0, 151.8, 154.1, 154.1; HRMS (ESI,* m*/*z*): calcd for C_27_H_32_N_2_O_2_Na, 439.2361 [M+Na]^+^; found: 439.2367.

*Urea Derivative* **4***.* A mixture of the urea derivative **3** (580 mg, 1.39 mmol), 3-bromo-1-propanol (280 mg, 2.09 mmol), and K_2_CO_3_ (1.15 g, 8.35 mmol) in DMF (13.9 mL) was stirred at 60 °C for 48 h and then the solvent was evaporated under reduced pressure. The residue was partitioned between CH_2_Cl_2_ (3 × 40 mL) and saturated brine (80 mL); the combined organic phases were dried (MgSO_4_) and concentrated. The residue was purified chromatographically (SiO_2_; EtOAc/hexanes, 4:6) to afford a white solid (350 mg, 53%). M.p. 226–228 °C; ^1^H-NMR (400 MHz, CD_3_COCD_3_): δ = 1.38 (s, 18H), 1.95 (quint,* J* = 6.4 Hz, 2H), 3.61 (t, *J* = 5.6 Hz, 1H), 3.70–3.76 (m, 2H), 4.07 (t, *J* = 6.4 Hz, 2H), 6.88 (d, *J* = 8.8 Hz, 2H), 7.42–7.47 (m, 5H), 7.57 (d, *J* = 8.8 Hz, 2H), 7.62 (d, *J* = 8.8 Hz, 2H), 7.93 (s, 1H), 8.12 (s, 1H); ^13^C-NMR (100 MHz, CD_3_COCD_3_): δ = 31.9, 33.5, 35.6, 59.2, 65.9, 115.5, 119.7, 121.5, 121.7, 121.8, 128.3, 133.8, 136.8, 140.4, 141.1, 151.9, 153.8, 155.8; HRMS (ESI,* m*/*z*): calcd for C_30_H_39_N_2_O_3_, 475.2961 [M+H]^+^; found: 475.2905.

*Isocyanate* **6**. A solution of triphosgene (383 mg, 1.29 mmol) in THF (35 mL) was added to a solution of **5** (826 mg, 2.58 mmol) and Et_3_N (1.48 mL, 10.61 mmol) in THF (35 mL) at 0 °C and then the mixture was stirred at room temperature for 3 h. The solids were filtered off and the filtrate concentrated under reduced pressure to afford a purple liquid (804 mg, 90%), which was used directly without further purification.

*[2]rotaxane* **1*** and Dumbbell-Shaped Molecule* **7**. A solution of the threadlike urea derivative **4** (350 mg, 0.737 mmol), BPX26C6 (770 mg, 1.85 mmol), and NaTFPB (1.64 g, 1.85 mmol) in CH_2_Cl_2_ (3.7 mL) was added to a solution of the isocyanate **6** [transformed from 826 mg (2.58 mmol) of **5**] and di-*n*-butyltin dilaurate (156 μL, 0.252 mmol) in CH_2_Cl_2_ (3.7 mL) and then the mixture was stirred at room temperature for 16 h. The solvent was evaporated under reduced pressure and the residue purified chromatographically (SiO_2_; EtOAc/hexanes, from 2:8 to 4:6) to afford the dumbbell-shaped molecule **7** as an off-white solid (60.8 mg, 10%). The fractions containing the [2]rotaxane **1** were collected and concentrated and the residue purified chromatographically (SiO_2_; CH_3_OH/CH_2_Cl_2_, 2:98) to afford the [2]rotaxane **1** as an off-white solid (270 mg, 30%). Data for the [2]rotaxane **1**: M.p. 112–114 °C; ^1^H-NMR (400 MHz, CD_3_CN): δ = 1.38 (s, 18H), 1.78 (quint,* J* = 6 Hz, 2H), 3.47–3.68 (m, 16H), 3.75 (s, 6H), 3.84 (t, *J* = 6.4 Hz, 2H), 3.90 (t, *J* = 5.2 Hz, 2H), 4.31 (d, *J* = 12 Hz, 4H), 4.31 (d, *J* = 12 Hz, 4H), 6.76–6.89 (m, 8H), 6.95 (d, *J* = 8.8 Hz, 4H), 6.99 (s, 8H), 7.13 (d, *J* = 8.8 Hz, 2H), 7.15 (s, 1H), 7.23 (d, *J* = 8.8 Hz, 2H), 7.26–7.34 (m, 3H), 7.42–7.47 (m, 3H), 7.50 (d, *J* = 8.4 Hz, 2H), 7.72 (s, 1H); ^13^C-NMR (100 MHz, CD_3_CN): δ = 30.0, 32.2, 36.1, 56.6, 62.6, 66.4, 70.2, 71.9, 74.3, 116.0, 116.1, 120.5, 121.2, 122.2, 122.4, 124.1, 126.9, 128.6, 129.8, 134.3, 134.8, 136.5, 138.6, 140.7, 141.9, 143.1, 145.1, 152.8, 153.5, 155.0, 155.7, 157.0 (one signal missing, possibly because of signal overlap). HRMS (ESI,* m*/*z*): calcd for C_75_H_88_N_4_O_12_Na, 1259.6296 [M+Na]^+^; found: 1259.6246. Data for dumbbell-shaped molecule **7**: M.p. 149–151 °C; ^1^H-NMR (400 MHz, CD_3_CN): δ = 1.36 (s, 18H), 2.07 (quint,* J* = 6.4 Hz, 2H), 3.74 (s, 6H), 4.06 (t, *J* = 6.0 Hz, 2H), 4.26 (t, *J* = 6.4 Hz, 2H), 6.80–6.86 (m, 6H), 6.89 (d, *J* = 8.8 Hz, 4H), 6.95 (d, *J* = 8.8 Hz, 2H), 7.21–7.29 (m, 3H), 7.34 (d, *J* = 9.2 Hz, 2H), 7.41–7.46 (m, 4H), 7.51 (d, *J* = 8.8 Hz, 2H), 7.54–7.65 (m, 3H); ^13^C-NMR (100 MHz, CD_3_CN): δ = 30.3, 32.2, 36.1, 56.6, 62.9, 66.3, 116.1, 116.3, 120.7, 121.6, 122.5, 122.6, 123.0, 123.6, 127.2, 129.0, 133.9, 137.5, 140.4, 141.6, 142.9, 145.9, 152.8, 154.6, 155.4, 156.4, 157.2 (one signal missing, possibly because of signal overlap). HRMS (ESI,* m*/*z*): calcd for C_51_H_56_N_4_O_6_, 820.4200 [M]^•+^; found: 820.4164.

*[2]rotaxane* **8**. Triisopropylsilyl trifluoromethanesulfonate (517 mg, 1.69 mmol) and DIEA (65.3 mg, 0.505 mmol) were added to a solution of the urea-containing threadlike molecule **4** (200 mg, 0.422 mmol), BPX26C6 (439 mg, 1.05 mmol), and NaTFPB (934 mg, 1.05 mmol) in CH_2_Cl_2_ (4.2 mL) and then the mixture was stirred at room temperature for 16 h. The solvent was evaporated under reduced pressure and the residue purified chromatographically (SiO_2_; EtOAc/hexanes, 4:6) to afford a yellow oil (120 mg, 27%). ^1^H-NMR (400 MHz, CD_3_CN): δ = 1.05–1.16 (m, 21H), 1.39 (s, 18H), 1.92–1.97 (m, 5H), 3.53–3.69 (m, 16H), 3.91 (t, *J* = 6.0 Hz, 2H), 4.06 (t, *J* = 6.2 Hz, 2H), 4.31 (s, 8H), 6.75 (d, *J* = 8.9 Hz, 2H), 6.94 (s, 8H), 6.96–7.00 (m, 3H), 7.09–7.15 (m, 3H), 7.42–7.49 (m, 5H); ^13^C-NMR (100 MHz, CD_3_CN): δ = 13.3, 19.0, 32.3, 34.0, 36.1, 61.2, 66.0, 70.2, 72.0, 74.3, 115.7, 121.8, 122.3, 122.3, 128.3, 129.6, 134.6, 135.7, 138.5, 140.9, 142.1, 152.6, 152.7, 155.3 ppm (two signals were missing, possibly because of overlap). HRMS (ESI): calcd for C_63_H_90_N_2_O_9_NaSi, *m*/*z* 1069.6313 [M+Na]^+^; found: 1069.6356.

### 3.2. UV–Vis Spectroscopy 

A solution of the [2]rotaxane **1** or the dumbbell-shaped molecule **7** was stirred with an excess of Zn powder in CH_3_CN for 30 min and then the solids were filtered off and the filtrate concentrated. UV–Vis spectra were recorded after adding an aliquot of NOBF_4_/CH_3_CN (3 × 10^−2^ M) into the solution of the [2]rotaxane **1** or the dumbbell-shaped molecule **7** (3 × 10^−4^ M). All solvents were freshly distilled and deoxygenated with Ar prior to use. A cell having a path length of 0.1 cm was used to acquire the spectra. All UV–Vis spectral measurements were performed using a Cary 50 spectrometer (Varian, Palo Alto, CA, USA) and a personal computer.

### 3.3. Electrochemistry 

CV was performed to determine the oxidation potentials of the [2]rotaxane **1** and the dumbbell-shaped molecule **7** (5 × 10^−4^M). The electrochemical instrumentation comprised a CHI 627C electrochemical analyzer (CH Instrument, Austin, TX, USA) and a conventional personal computer. The conventional three-electrode configuration featured a Pt disk (0.07 cm^2^) electrode as the working electrode, a Pt counter electrode, and a Ag/AgCl electrode as the reference electrode. The reference electrode was calibrated (using Fc/Fc^+^) before and after each experiment. TBAPF_6_ (0.1 M) was employed as the supporting electrolyte. The measurements were performed under Ar in anhydrous solvents that had been freshly distilled prior to use. The triphenylamine-containing [2]rotaxane **1** and the dumbbell-shaped molecule **7** were treated with Zn powder prior to measurement, as described above. All samples were deoxygenated with Ar prior to measurement.

### 3.4. Spectroelectrochemistry 

A quartz cuvette having a path length of 0.1 cm was used for spectroelectrochemical measurements. Pt gauze (100 mesh) was employed as an optically transparent electrode. The counter and reference electrodes were Pt wire and Ag/AgCl electrode, respectively. The UV–Vis and CV measurements were performed using the same instrument described above. Samples for spectroelectrochemistry were prepared in the same manner as those for CV measurements.

### 3.5. Electron Paramagnetic Resonance Spectroscopy 

Electron paramagnetic resonance spectra of equimolar mixtures (3 mM) of NOBF_4_ and the [2]rotaxane **1** or the dumbbell-shaped molecule **7** in CH_3_CN were measured at 298 K using an ELEXSYS E580 spectrometer (Bruker, Billerica, MA, USA).

## 4. Conclusions

We have demonstrated that the Na^+^ ion–assisted recognition of urea derivatives by BPX26C6 allows the construction of a redox-controllable [2]rotaxane-type molecular switch based on two originally very weakly interacting host/guest systems. Using NOBF_4_ to oxidize the triarylamine terminus into a corresponding radical cation, the macrocyclic component was attracted toward the adjacent carbamate station; subsequent addition of Zn powder moved the macrocyclic component back to its original state. We believe that such a recognition system will be valuable for the development of molecular switches and functional materials having novel designs and structures; by allowing the construction of interlocked molecules featuring relatively weak host/guest interactions, various chemically, electrochemically, and photochemically active functionalities can be installed in these (macro)molecules to serve as stations during the switching process.

## Supplementary Materials

Supplementary materials can be accessed at: http://www.mdpi.com/1420-3049/20/02/1775/s1.
