# Supplementary material for: A Redox-Controllable Molecular Switch Based on Weak Recognition of BPX26C6 at a Diphenylurea Station
Source: Molecules. 2015 Jan 22;20(2):1775–87. doi: 10.3390/molecules20021775 (PMC6272467; doi:10.3390/molecules20021775)
Supplement: Supplementary file 1 [file molecules-20-01775-s001.pdf]

Supplementary Materials

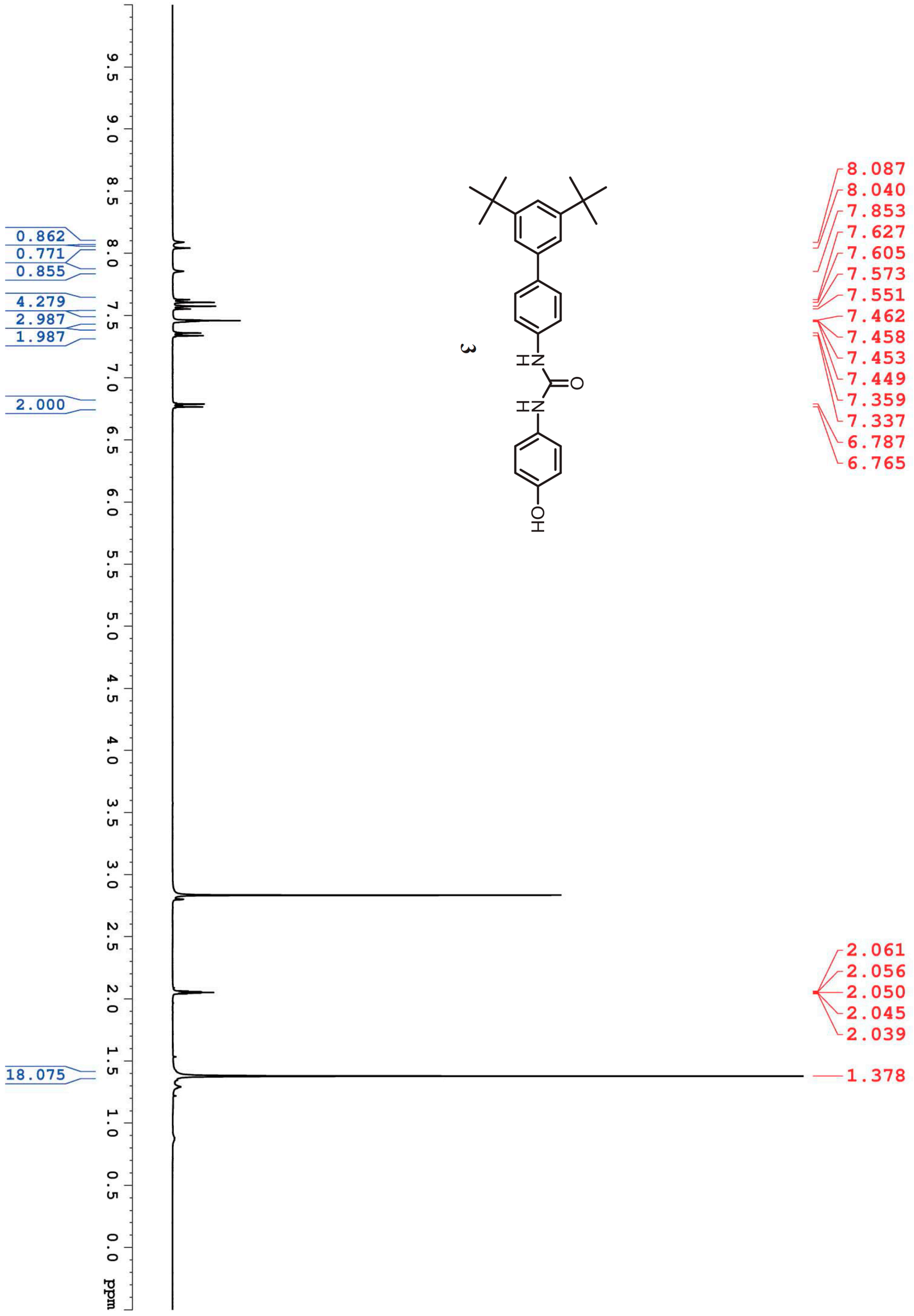

Figure S1. *Cout.*

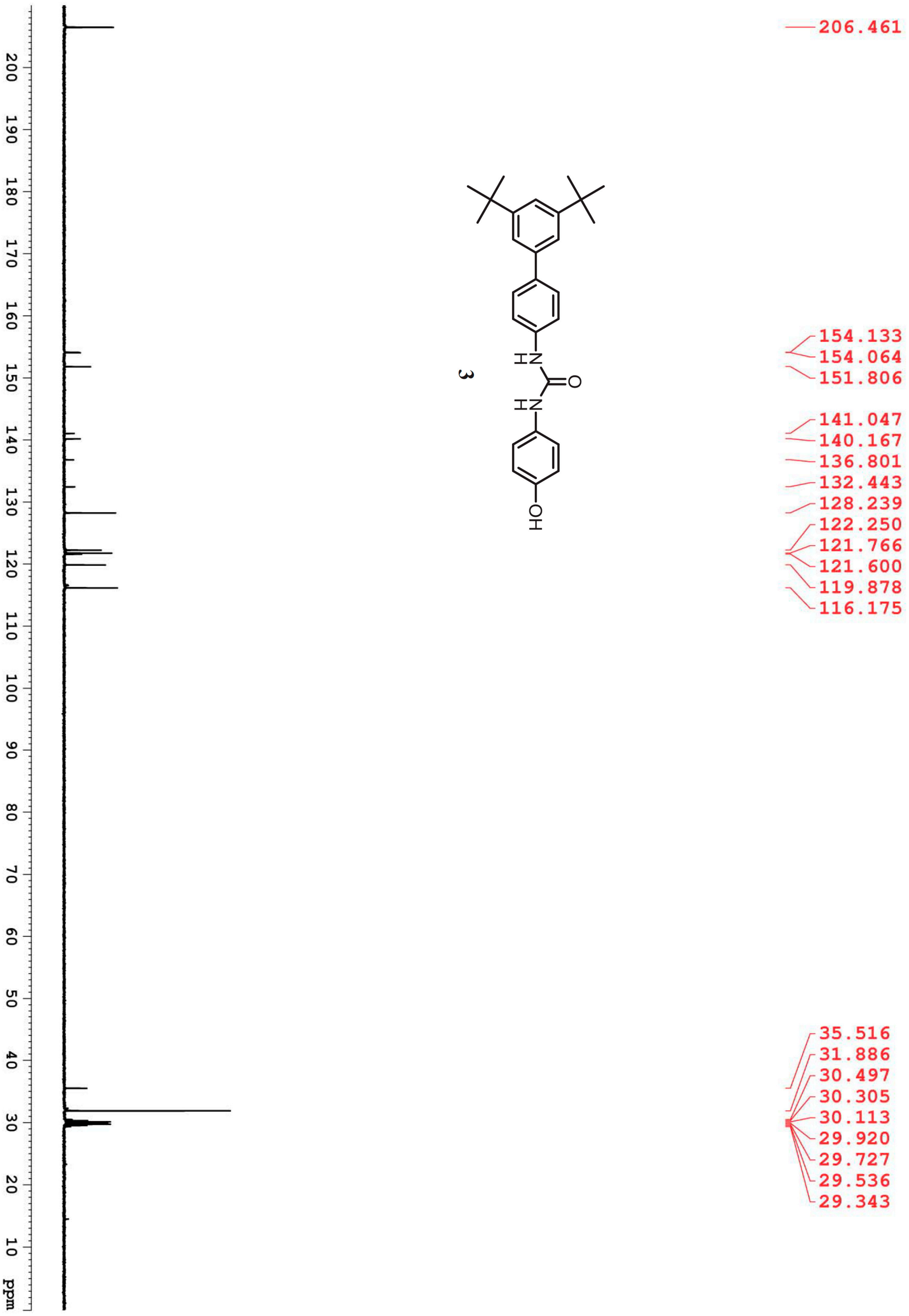

Figure S1. ^{1}H and ^{13}C-NMR Spectra of the Urea **3**.

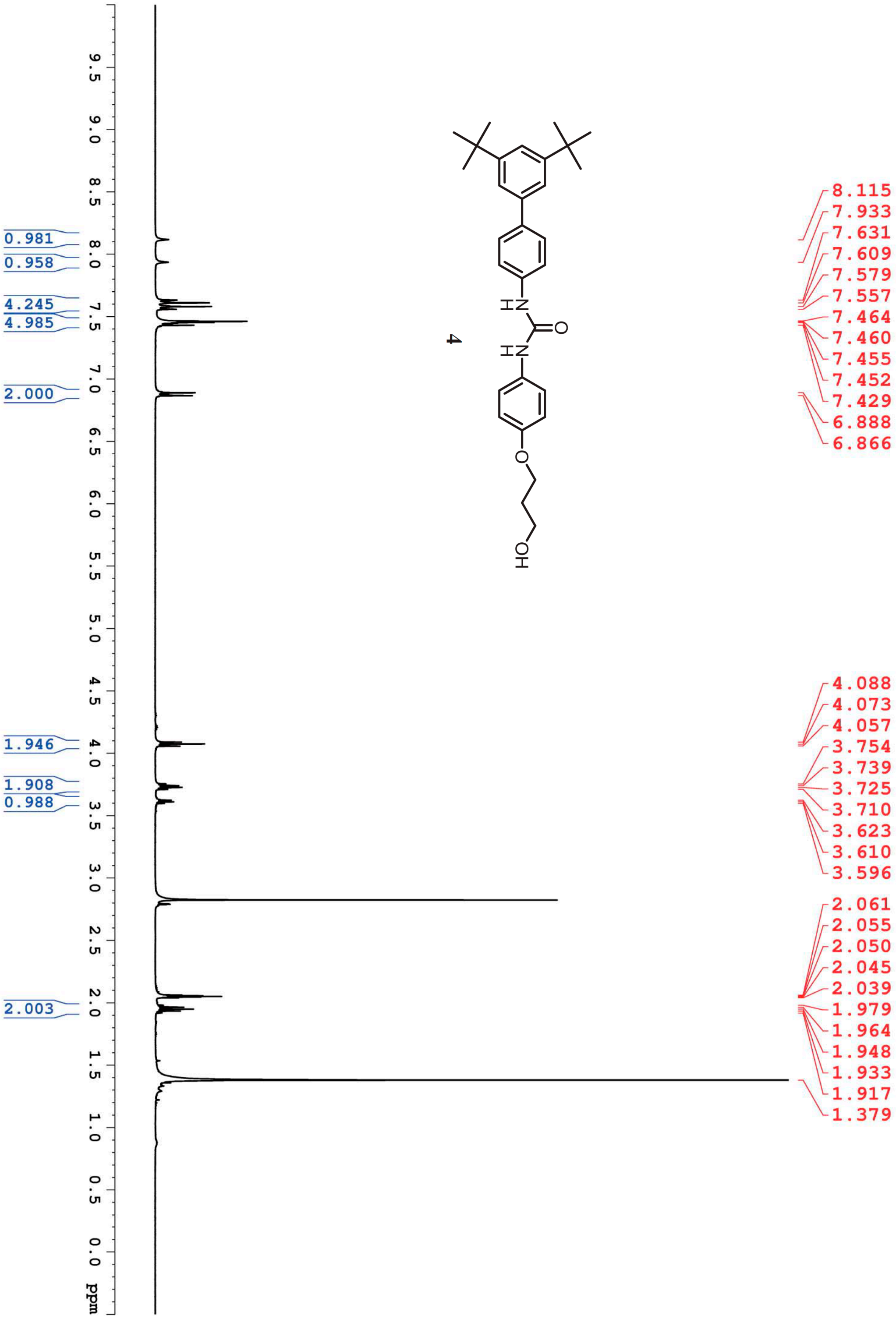

Figure S2. *Cout.*

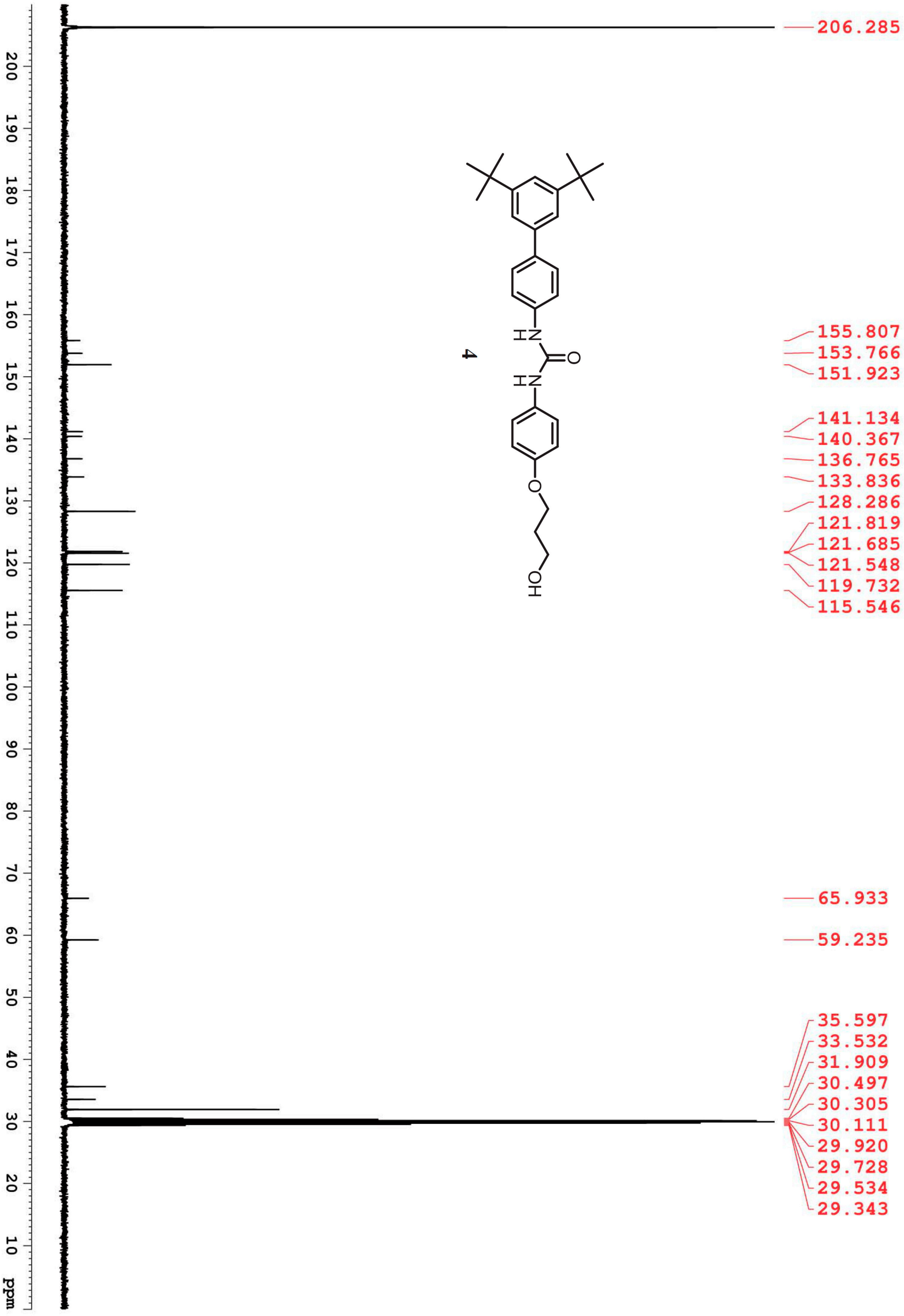

Figure S2. ^{1}H and ^{13}C-NMR Spectra of the Urea **4**.

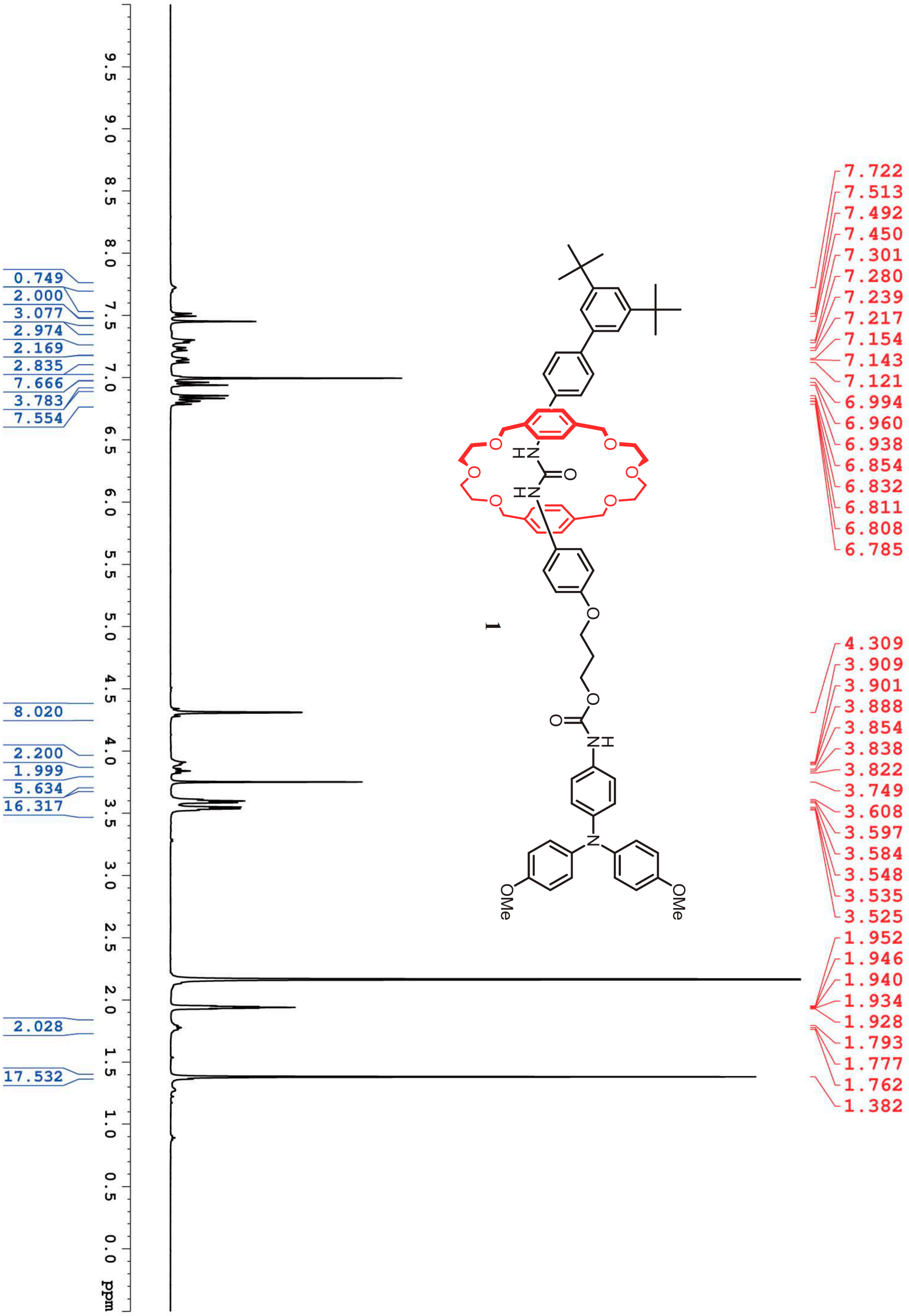

Figure S3. *Cout.*

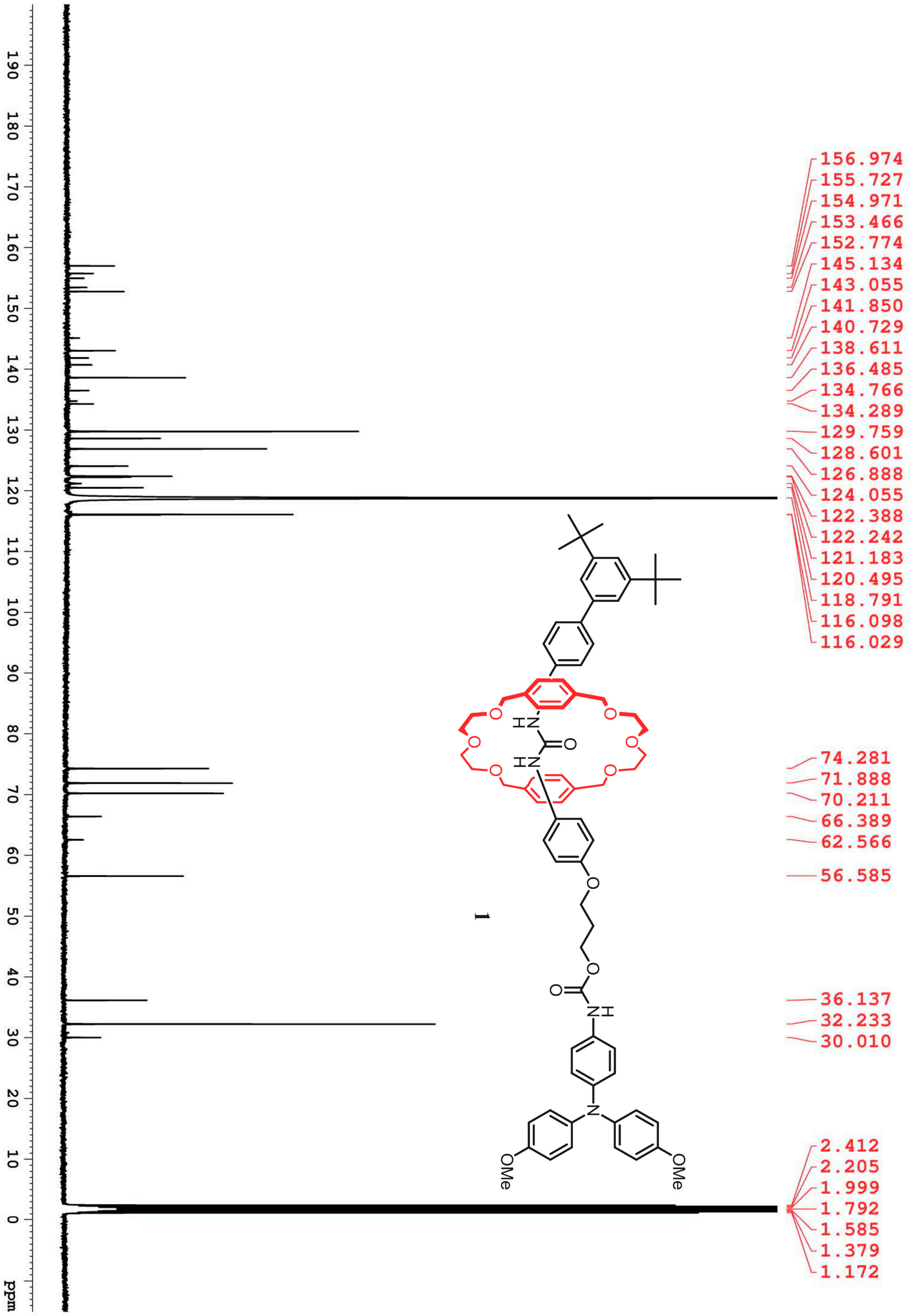

Figure S3. ^{1}H and ^{13}C-NMR Spectra of the [2]Rotaxane **1**.

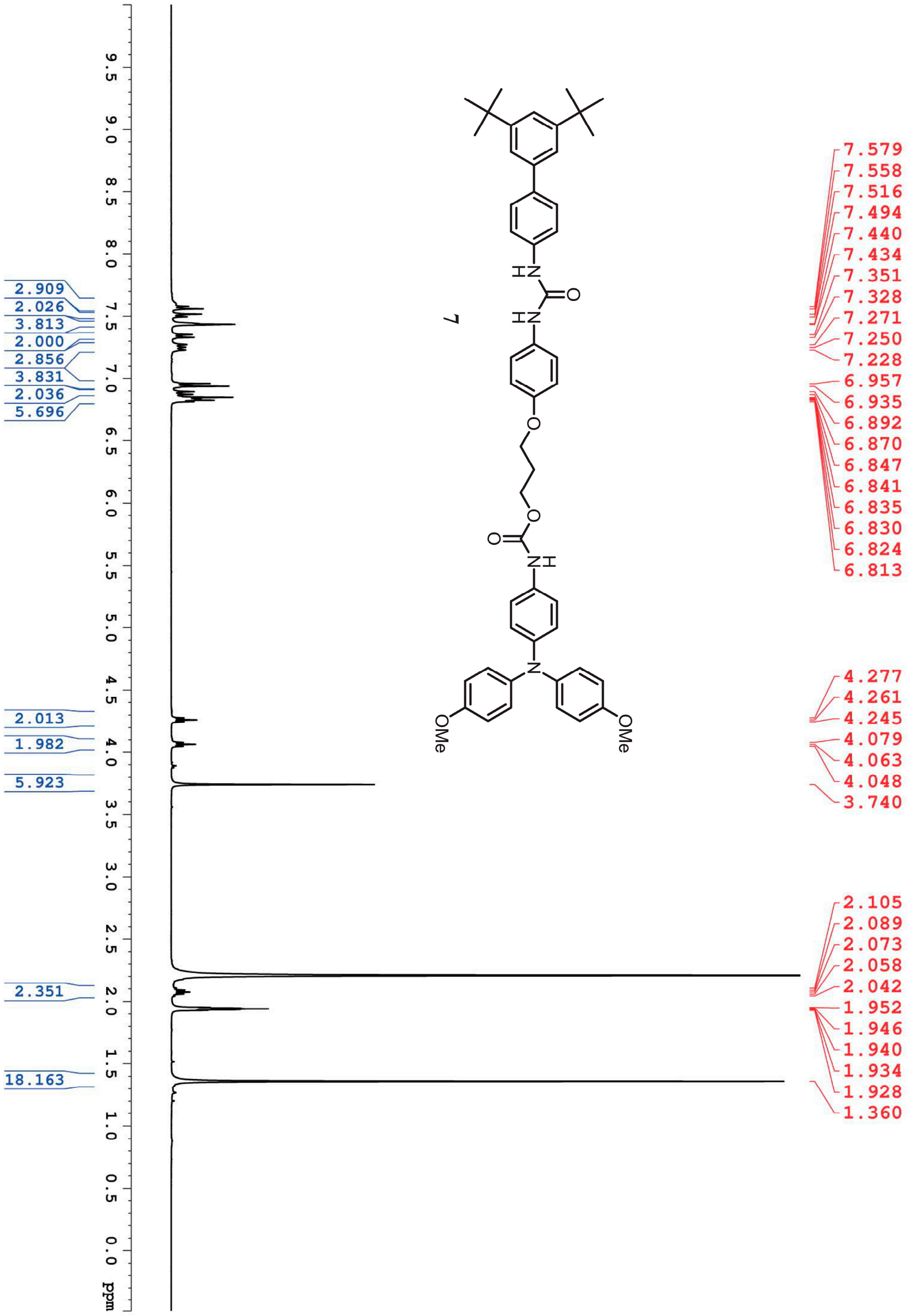

Figure S4. *Cout.*

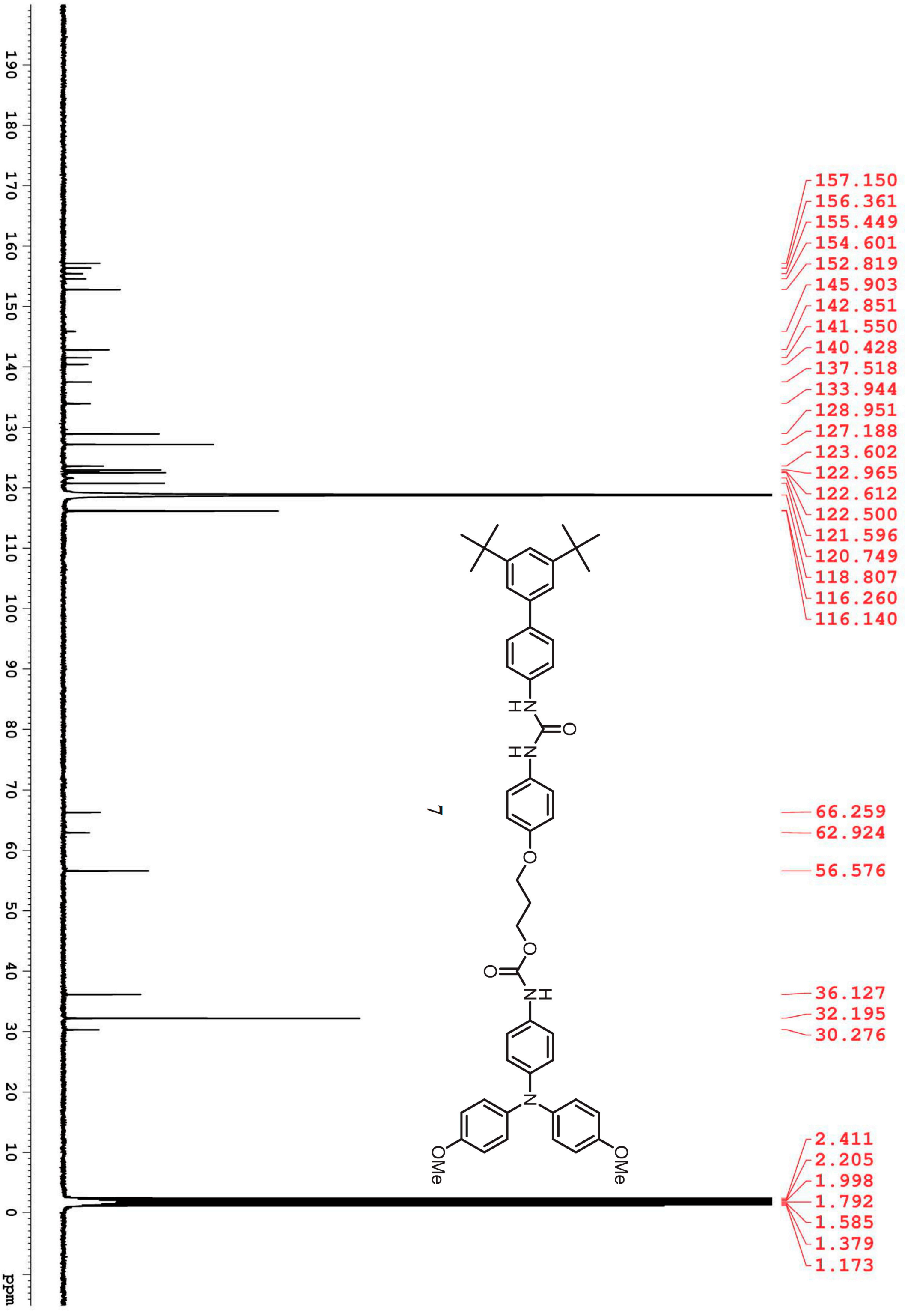

Figure S4. ^{1}H and ^{13}C-NMR Spectra of the Dumbbell **7**.

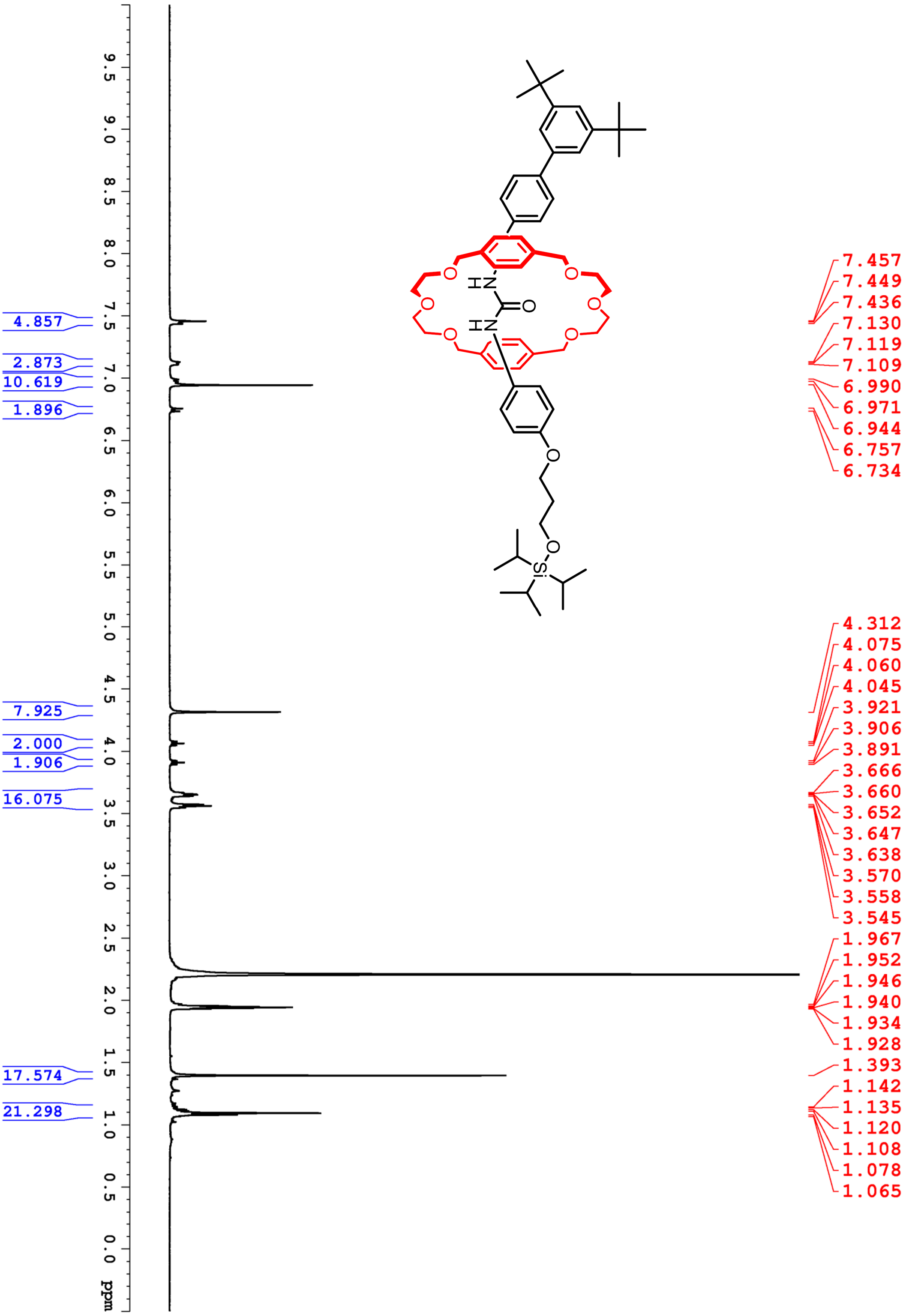

Figure S5. *Cout.*

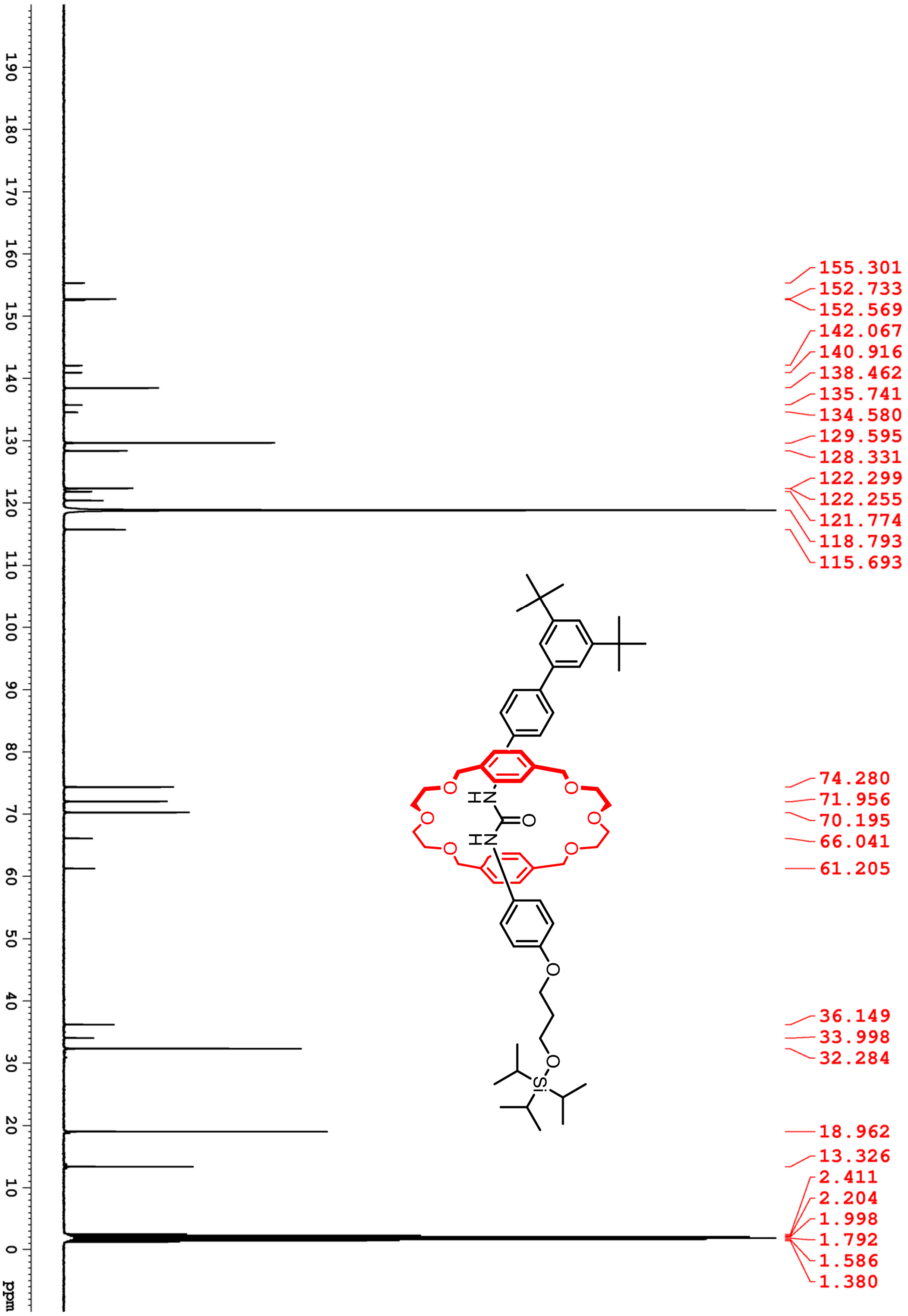

Figure S5. ^{1}H and ^{13}C-NMR Spectra of the [2]Rotaxane **8**.

COSY Spectrum (400 MHz, CD_3CN, 298 K) of [2]rotaxane **1**

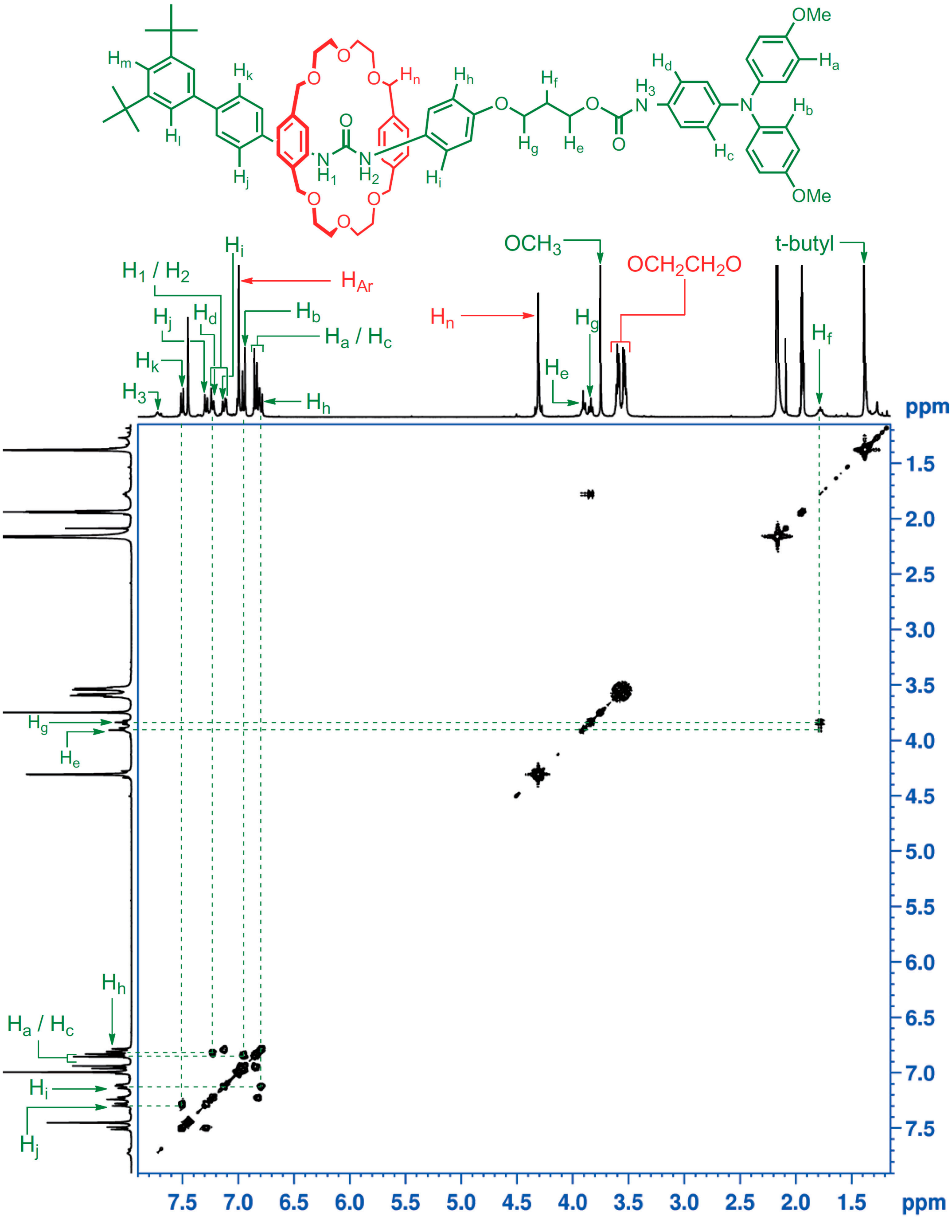

Figure S6. *Cout.*

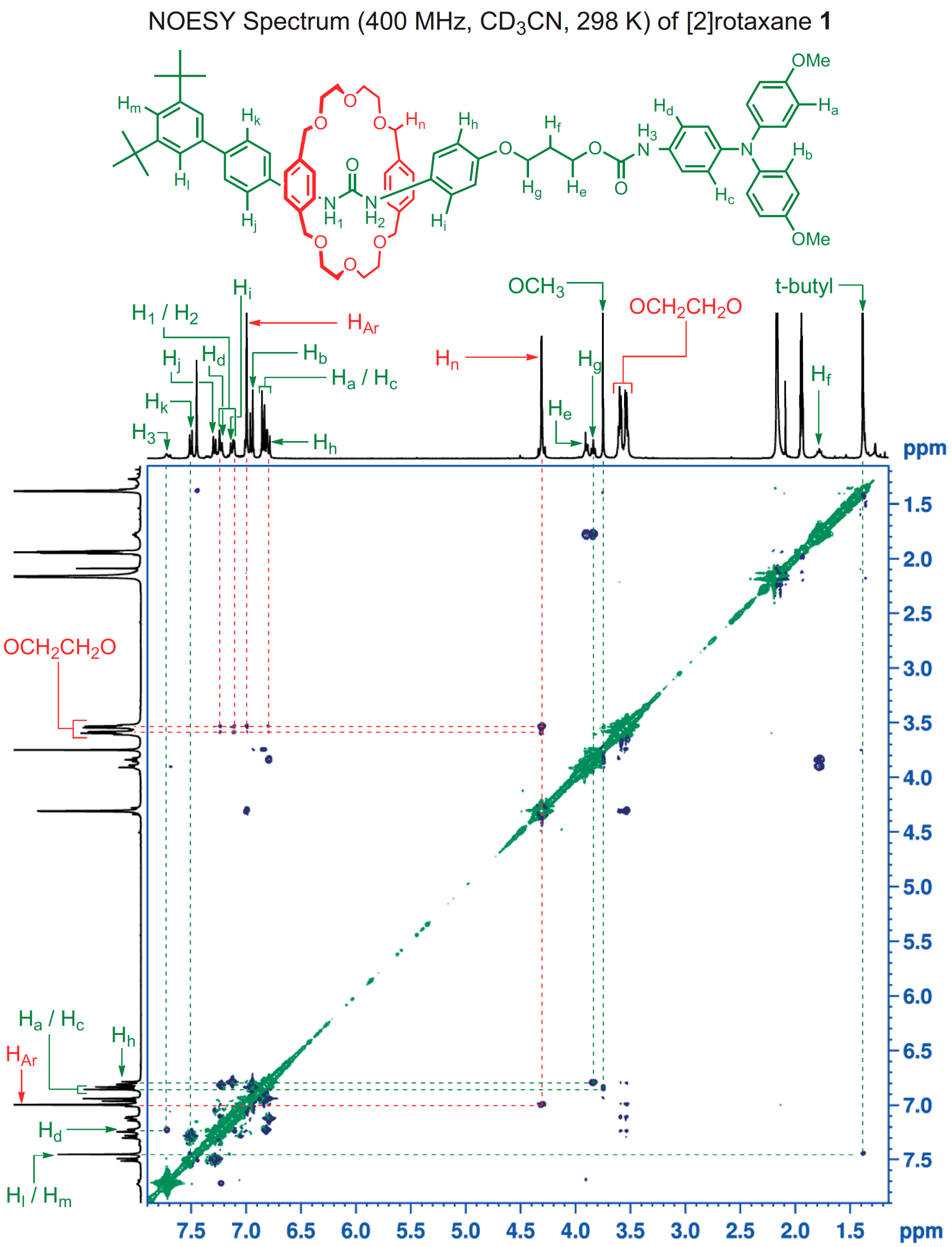

Figure S6. 2D COSY and NOSEY Spectra of the [2]Rotaxane **1**.

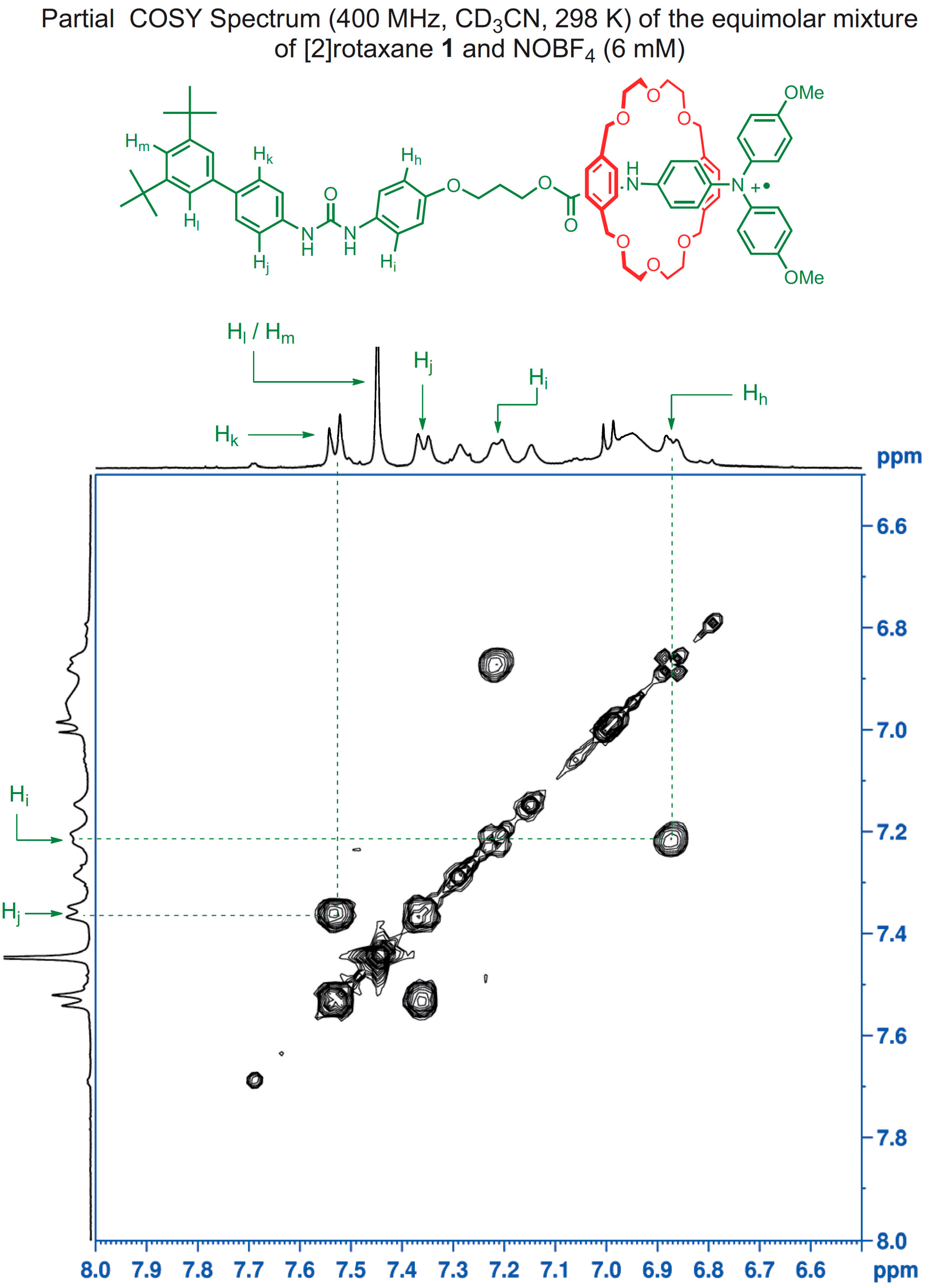

Figure S7. Partial 2D COSY Spectra of the equimolar mixture of [2]Rotaxane **1** and $NOBF_4$ (6 mM).
